# Postoperative delirium after lung resection for primary lung cancer: Risk factors, risk scoring system, and prognosis

**DOI:** 10.1371/journal.pone.0223917

**Published:** 2019-11-18

**Authors:** Kazuki Hayashi, Makoto Motoishi, Satoru Sawai, Kanna Horimoto, Jun Hanaoka

**Affiliations:** 1 Division of General Thoracic Surgery, Department of Surgery, Shiga University of Medical Science, Shiga, Japan; 2 Department of Thoracic Surgery, Mitsubishikyoto Hospital, Kyoto, Japan; 3 Department of Thoracic Surgery, National Hospital Organization Kyoto Medical Center, Kyoto, Japan; Baylor College of Medicine, UNITED STATES

## Abstract

Delirium is a common post-surgical complication, but few studies have examined postoperative delirium following lung cancer surgery. The purpose of this study was to clarify the risk factors of postoperative delirium, to construct a useful scoring system, and to clarify the relationship between delirium and prognosis after lung cancer surgery. We retrospectively analyzed data from 570 patients who underwent surgery for primary lung cancer. Logistic regression analysis was used to determine the effects of various factors on the onset of delirium. Kaplan–Meier analysis was performed to determine the relationship between delirium and prognosis. Postoperative delirium occurred in 6.7% of the patients. Three risk factors were identified, and the risk scores were determined as follows: 2×(cerebrovascular disease history) + 1×(squamous cell carcinoma) + 1×(age older than 75 years). Scores 0–1 denoted low risk, 2 denoted intermediate risk, and 3–4 denoted high risk. Additionally, we found that patients who developed delirium had significantly shorter overall survival. However, there was no difference in the frequency between cancer-related death and non-cancer related death when comparing the delirium and non-delirium groups. We identified the risk factors, i.e., cerebrovascular disease history, squamous cell carcinoma, and age older than 75 years, that determine the onset of delirium after lung cancer surgery and constructed a useful scoring system. In addition, although the prognosis of the delirium group was poor, the factor that determines prognosis may not be cancer per se but vulnerability in the patient background.

## Introduction

Delirium, an acute and transient confusional state, is defined as a disturbance in attention and awareness that develops rapidly and tends to fluctuate [1]. Delirium arising after surgery is reportedly related to increased mortality and prolonged hospitalization, which can lead to more serious complications [2, 3]. Postoperative delirium is common, and several studies have investigated the risk factors for developing this condition [2–14]. Following thoracic surgery, 5–16% of patients have been reported to develop delirium [2,14,15]. However, few studies have examined the development of delirium after surgery for pulmonary malignancy [16, 17]. Older age is frequently considered a risk factor for postoperative delirium, but it has been reported that patients who develop delirium after thoracic surgery are mainly in their 50s [14, 15]. Conversely, primary lung cancer is prevalent in older age groups. Therefore, it would be meaningful to clarify whether older age is a risk factor for developing delirium among patients with lung cancer. Recently, surgery for lung cancer has been shifting from thoracotomy to minimally-invasive surgical procedures. It remains undetermined whether there are differences in the onset of delirium depending on the surgical procedure. In this study, we attempted to address these clinical questions. Moreover, if the associated risk factors are clarified, a useful method to predict the onset of delirium can be constructed.

No previous study has reported on the relationship between onset of delirium and prognosis after surgery for primary lung cancer. This study aimed to clarify the risk factors related to the onset of delirium after primary lung cancer surgery and to construct a clinically useful scoring system. Another purpose was to clarify the relationship between the onset of delirium and prognosis.

## Material and methods

### Data collection

Between December 2006 and January 2017, 590 patients with lung cancer underwent complete resection at the Kyoto Medical Center. Patients who underwent multiple surgeries for metachronous multiple lung cancer were excluded (n = 20), and we examined data from 570 consecutive patients.

The patients’ medical histories were reviewed retrospectively from the hospital records. Patient information is summarized in Tables 1, 2, 3 and 4. Cardiovascular disease included myocardial infarction, angina pectoris, cardiovalvular disease, atrial fibrillation, arteriosclerosis obliterans, and aortic disease. Cerebrovascular disease included cerebral infarction, cerebral hemorrhage, carotid artery stenosis, and cerebral aneurysm. Psychiatric disorders included depression and anxiety neurosis. There were no patients with schizophrenia in our patient group. The above comorbidities were diagnosed by specialists in each area. The study protocol was approved by the Kyoto Medical Center ethics committee. All patients were provided the opportunity to consent or opt-out of the study during recruitment.

**Table 1:** Patient demographics and preoperative factors.

Variable	Overall (n = 570)	Delirium (n = 38)	Non-delirium (n = 532)	*P* value
Median age, years (range)	70 (35–88)	75.5 (62–88)	70 (35–88)	<0.001^†^
Male sex, n (%)	369 (64.7)	32 (84.2)	337 (63.4)	0.009^††^
Body mass index, n (%)				0.22^††^
<18.5	41 (7.2)	5 (13.2)	36 (6.8)	
18.5–24.9	396 (69.5)	27 (71.1)	369 (69.4)	
>25	133 (23.3)	6 (15.7)	127 (23.8)	
Brinkman index (range)	750 (0–4000)	900 (0–3600)	700 (0–4000)	0.008^†^
Pathological stage, n (%)				0.67^††^
0–I	378 (66.3)	24 (63.2)	354 (66.5)	
II–IV	192 (33.7)	14 (36.8)	178 (33.5)	
Histologic structure, n (%)				<0.001^††^
Adenocarcinoma	356 (62.4)	13 (34.2)	343 (64.5)	
Squamous cell carcinoma	160 (28.1)	21 (55.3)	139 (26.1)	
Others	54 (9.5)	4 (10.5)	50 (9.4)	
ASA-PS, n (%)				<0.001^**†††**^
1, 2	506 (88.8)	26 (68.4)	480 (90.2)	
3+	64 (11.2)	12 (31.6)	52 (9.8)	
Preoperative comorbidity, n (%)				
Hypertension	160 (28.1)	10 (26.3)	150 (28.2)	0.80^††^
Diabetes mellitus	66 (11.6)	7 (18.4)	59 (11.1)	0.19^**†††**^
Cardiovascular disease	65 (11.4)	9 (23.7)	56 (10.5)	0.03^**†††**^
Cerebrovascular disease	30 (5.2)	8 (21.1)	22 (4.1)	<0.001^**†††**^
Psychiatric disorder	7 (1.23)	1 (2.6)	6 (0.88)	0.39^**†††**^
Dementia	6 (1.05)	3 (7.89)	3 (0.56)	0.005^**†††**^
Alcohol abuse	1 (0.18)	1 (2.6)	0 (0.00)	0.07^**†††**^
Other malignancy	135 (23.7)	11 (28.9)	124 (23.3)	0.43^††^
Pulmonary emphysematous change	257 (45.2)	25 (65.8)	232 (43.7)	0.008^††^
Preoperative blood measurements (range)				
Total protein (g/dl)	7.1 (5.2–9.9)	7.1 (5.2–8.6)	7.1 (5.2–9.9)	0.76^†^
Albumin (g/dl)	4.2 (0.4–5.3)	3.8 (0.4–4.9)	4.2 (2.1–5.3)	<0.001^†^
Lymphocytes (10^3^/μl)	1.6 (0.4–6.5)	1.6 (0.9–3.7)	1.6 (0.4–6.5)	0.35^†^
Median PNI (range)	49.5 (4.5–77.5)	47.5 (8.5–61.5)	49.5 (4.5–77.5)	0.69^†^
Sodium (mEq/l)	140 (122–148)	140 (131–143)	140 (122–148)	0.66^†^
Potassium (mEq/l)	4.3 (3.0–5.5)	4.2 (3.6–5.4)	4.3 (3.0–5.5)	0.66^†^
Glucose (mg/dl)	102 (57–318)	107 (71–241)	101 (57–318)	0.13^†^
Hemoglobin (g/dl)	13.2 (8.4–16.9)	13.1 (9.9–16.9)	13.2 (8.4–16.9)	0.34^†^
MCV (fL)	95 (41.8–113.8)	97.3 (85–113.8)	94.9 (41.8–113.6)	0.002^†^
CRP (mg/dl)	0.09 (0–23.27)	0.125 (0–7.91)	0.09 (0–23.27)	0.04^†^
Preoperative pukmonary function test (range)				
%VC (%)	105.3 (45.6–162.9)	96.5 (59.0–131.7)	105.6 (45.6–162.9)	0.02^†^
%FEV1 (%)	102.3 (38.8–200.9)	102.4 (58.3–186.0)	102.3 (38.8–200.9)	0.66^†^
FEV1%	75.0 (40.8–100.0)	73.1 (49.5–92.5)	75.1 (40.8–100.0)	0.29^†^

%VC, percent vital capacity; %FEV1, percent forced expiratory volume in 1 s; FEV1%

ASA-PS, American Society of Anesthesiologists physical status; CRP, C-reactive protein; MCV, mean corpuscular volume; PNI, prognostic nutritional index; FEV1%, forced expiratory volume in 1 s

†, Mann–Whitney *U*-test

††, Pearson's chi-squared test

†††, Fisher's exact test

**Table 2:** Intraoperative factors.

Variable	Overall (n = 570)	Delirium (n = 38)	Non-delirium (n = 532)	*P* value
Operated side, n (%)				0.14^†^
Left	229 (40.2)	11 (28.9)	218 (41.0)	
Right	341 (59.8)	27 (71.1)	314 (59.0)	
Approach, n (%)				0.51^†^
VATS	329 (57.7)	20 (52.6)	309 (58.1)	
Open	241 (42.3)	18 (47.4)	223 (41.9)	
Surgical procedure, n (%)				0.85^†^
Wedge resection	101 (17.7)	8 (21.1)	93 (17.5)	
Segmentectomy or lobectomy	410 (71.9)	26 (68.4)	384 (72.2)	
Pneumonectomy or extended operation	59 (10.4)	4 (10.5)	55 (10.3)	
Length of procedure, min (range)	264 (44–700)	267.5 (56–487)	264.0 (44–700)	0.69^††^
Bleeding, ml (range)	70 (5–1820)	70 (5–1820)	70 (5–1640)	0.87^††^
Intraoperative infusion volume, ml (range)	1800 (100–6300)	1800 (400–5400)	1800 (100–6300)	0.68^††^
Epidural anesthesia, n (%)	484 (84.9)	29 (84.9)	455 (85.5)	0.13^†^

VATS, video-assisted thoracic surgery

†, Pearson's chi-squared test

††, Mann–Whitney U-test

**Table 3:** Postoperative factors.

Variable	Overall (n = 570)	Delirium (n = 38)	Non-delirium (n = 532)	*P* value
Admission to ICU, n (%)	479 (84.0)	32 (84.2)	447 (84.0)	1.0^†^
Length of ICU stay, days (range)	1 (0–3)	1 (0–3)	1 (0–2)	0.62^††^
Postoperative hospital stay, days (range)	10 (3–91)	10 (3–58)	10 (3–91)	0.96^††^
Postoperative complications, n (%)				
Overall incidence	131 (22.9)	14 (39.5)	116 (21.8)	0.03^†††^
Air leakage over 7 days	33 (5.8)	1 (2.6)	32 (6.0)	
Pneumonia	18 (3.2)	4 (10.5)	14 (2.6)	
Bronchial fistula	4 (0.7)	1 (2.6)	3 (0.6)	
Pyothorax	2 (0.4)	1 (2.6)	1 (0.2)	
Atelectasis	2 (0.4)	-	2 (0.4)	
Chylothorax	7 (1.3)	-	7 (1.3)	
Hemothorax	2 (0.4)	-	2 (0.4)	
Wound infection	18 (3.2)	4 (10.5)	14 (2.6)	
Acute exacerbation of interstitial pneumonia	1 (0.2)	1 (2.6)	-	
Atrial fibrillation	21 (3.7)	2 (5.3)	19 (3.6)	
Other	28 (4.9)	2 (5.3)	26 (4.9)	
Death within 30 days postoperatively, n (%)	7 (1.2)	4 (10.5)	3 (0.7)	< 0.001^†^

ICU, intensive care unit

†, Fisher's exact test

††, Mann-Whitney U-test

†††, Pearson's chi-squared test

**Table 4:** Causes of death within 30 days postoperatively.

Complications	Overall (n = 7)	Delirium (n = 4)	Non-delirium (n = 3)
Pneumonia	2	1	1
Bronchial fistula	3	1	2
Pyothorax	1	1	-
Acute exacerbation of interstitial pneumonia	1	1	-

### Perioperative management

After surgery, a thoracic drainage tube was used to confirm the absence of postoperative bleeding or lung fistula in all patients. Epidural anesthesia was routinely used for pain relief (0.75% ropivacaine hydrochloride hydrate). In cases involving impaired hemostatic function, intravenous patient-controlled analgesia was implemented. Non-steroidal anti-inflammatory drugs or acetaminophen was orally administered from the morning after surgery. Postoperative patients entered the intensive care unit (ICU) and returned to the surgical ward the following morning, unless their condition was not sufficiently stable.

### Diagnosis of delirium

Delirium was diagnosed by appropriate doctors based on the Diagnostic and Statistical Manual of Mental Disorders-IV-TR (DSM-IV-TR) or Diagnostic and Statistical Manual of Mental Disorders-V (DSM-V) depending on the time of recruitment in the general ward [18, 19] and on the Confusion Assessment Method for the ICU (CAM-ICU) in the ICU, which has been proven to be accurate [20–22]. Patients with delirium were treated with antipsychotics and sedatives, such as dexmedetomidine, as appropriate.

### Postoperative follow up

Postoperative follow up was performed every 3 months for the first 3 years, and every 6 months from the 4th year. Chest computed tomography was performed every 6 months and positron emission tomography and brain magnetic resonance imaging were performed annually. We defined postoperative recurrence of lung cancer as image-wise confirmed recurrence. The recurrence date was defined as the date on which image recurrence was confirmed. The length of overall survival (OS) was defined as the period from surgical resection to death or from surgical resection to the last follow up. The disease-free survival (DFS) period was defined as the period from surgery to the date when recurrence was confirmed.

### Statistical analysis

Statistical analyses were performed using SPSS statistical software (version 25.0.0.1, IBM, Tokyo, Japan). Normality controls showed that the variables were non-normally distributed and thus were analyzed with the Mann–Whitney U test. Categorical variables were compared using the chi-squared or Fisher’s exact test. Continuous data are presented as mean ± SD unless otherwise noted. Variables considered important in past reports and variables of interest for this group of patients were selected and logistic regression analysis was performed. The formula for calculating the risk score was derived using odds ratio values from the regression model.

Additionally, in order to investigate whether there was a difference in prognosis after lung cancer surgery depending on the presence or absence of delirium, estimation of DFS and OS was calculated by the Kaplan–Meier method, and the curves were compared using a log-rank test. A probability value less than 0.05 was considered statistically significant. The analysis with three variables was adjusted by Bonferroni’s method, and the probability value was considered significant at less than 0.016.

## Results and discussion

Of the 570 patients, 369 were male. The median overall age was 70 years (range, 35–88 years). Postoperative delirium occurred in 38 cases (6.7%), and the onset of the delirium ranged from day 0 to day 2 after surgery (mean, 0.76 ± 0.75 days). The duration of delirium ranged from 1 day to 30 days (mean, 3.6 ± 4.89 days). The median age of the delirium group was 75.5 years (range, 62–88 years) (Table 1). The median age of the non-delirium group was 70 years (range, 35–88 years, *P* < 0.001). No intraoperative factor was observed to affect delirium after surgery (Table 2). There were no differences between the groups during the ICU admission period or postoperative hospital days. However, the number of postoperative complications and 30-day postoperative mortality were significantly greater in the group with delirium (*P* = 0.03 and *P* < 0.001, respectively) (Tables 3 and 4).

**Fig 1:**
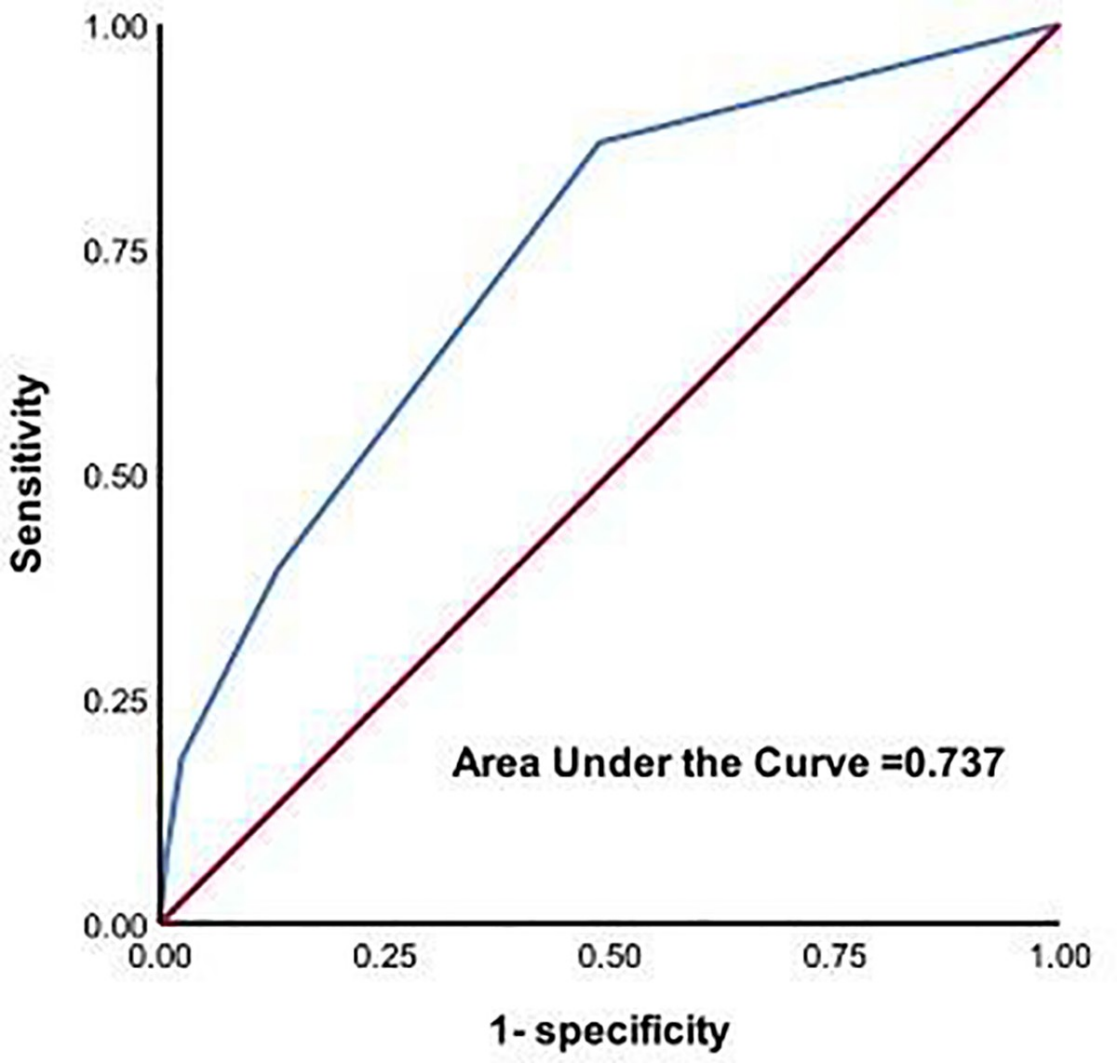
The receiver operator characteristic curve for the predictive value of the risk score. The risk score was moderately discriminatory with a c-index of 0.737.

**Fig 2:**
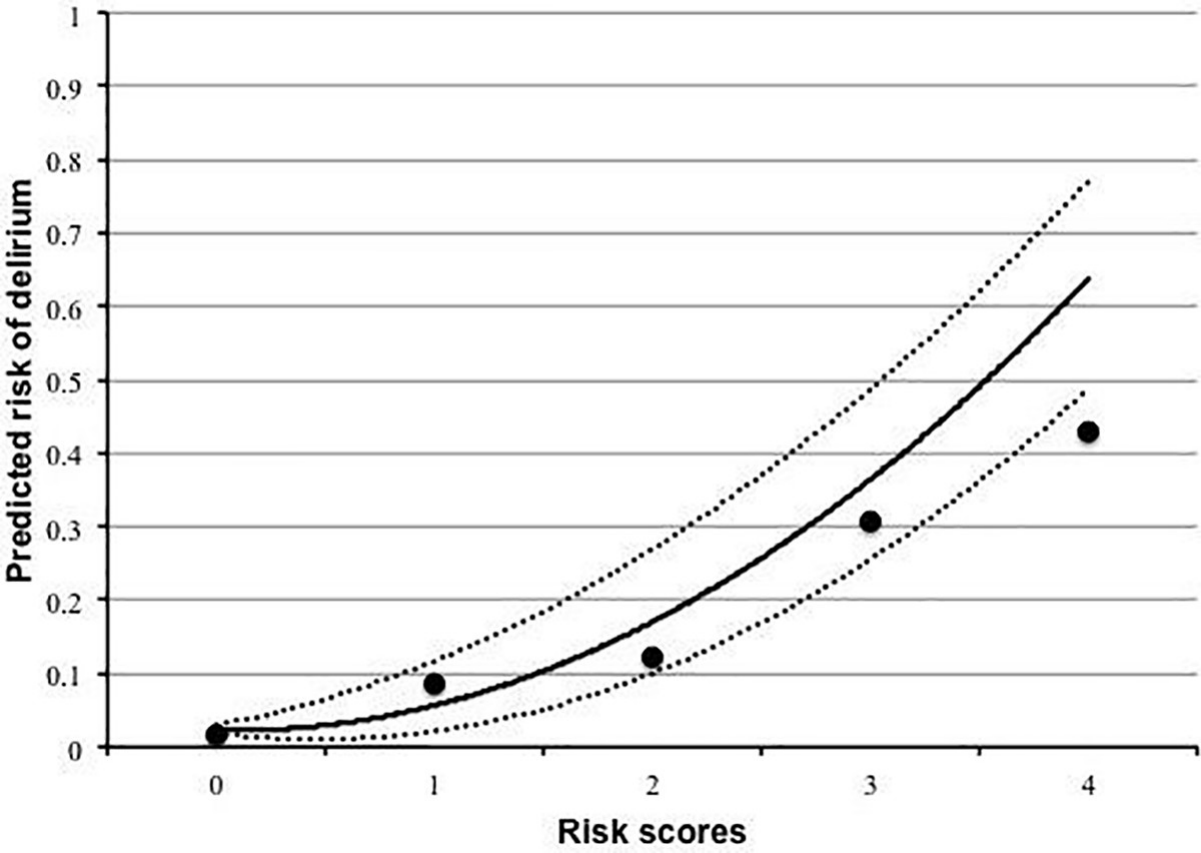
The relationship between risk score and predicted probability. Dots represent observed delirium (%), the curve, predicted delirium (%); the dotted curves, the 95% confidence interval; the horizontal axis, the risk scores.

Table 1 shows factors related to delirium after lung cancer surgery obtained by univariate analysis. Based on logistic regression analysis, history of cerebrovascular disease, squamous-cell carcinoma, and age older than 75 years were independent risk factors of postoperative delirium. Estimated odds ratios and confidence intervals were calculated (Hosmer–Lemeshow test: *P* = 0.280) (Table 5). Based on the regression coefficients, the calculation formula of the risk score for postoperative delirium was as follows: 1.600×(cerebrovascular disease history) +1.113×(squamous cell carcinoma) +0.871×(age older than 75 years). According to the weight of each variable, the modified formula for calculating the risk score was as follows: 2×(cerebrovascular disease history) +1×(squamous cell carcinoma) +1×(age older than 75 years). The receiver operating characteristic curve was as shown in Fig 1 and the c-index was 0.738, so this risk score system was moderately discriminatory. In addition, the relationship between the risk score and the number of patients and the probability of each are shown in Fig 2 and Table 6. According to the probability of delirium prediction, we divided patients into low-risk, intermediate-risk, and high-risk groups.

**Table 5:** Results of logistic regression analysis.

Variable	Groups	B	Exp (B)	95% CI	*P* value
Age	<75 versus ≥75	0.871	0.419	0,210–0.833	0.013
Histologic structure	others versus squamous cell carcinoma	1.113	0.329	0.165–0.653	0.002
Cerebrovascular disease	− versus +	1.600	0.262	0.079–0.513	0.001

B, regression coefficient; CI, confidence interval

**Table 6:** Distribution of the patients and their predicted risk of delirium.

Score	Number of patients	Predicted incidence of delirium (%)	95% CI	Observed incidence of delirium (%)	Patient's risk
0	277	2.1	1.2–3.7	5 (1.8)	Low risk
1	208	6.1	3.5–10.4	18 (8.7)	
2	65	16.3	9.8–25.9	8 (12.3)	Intermediate risk
3	13	36.9	24.5–51.3	4 (30.8)	High risk
4	7	63.7	49.3–76.0	3 (42.9)	

CI, confidence interval

**Fig 3:**
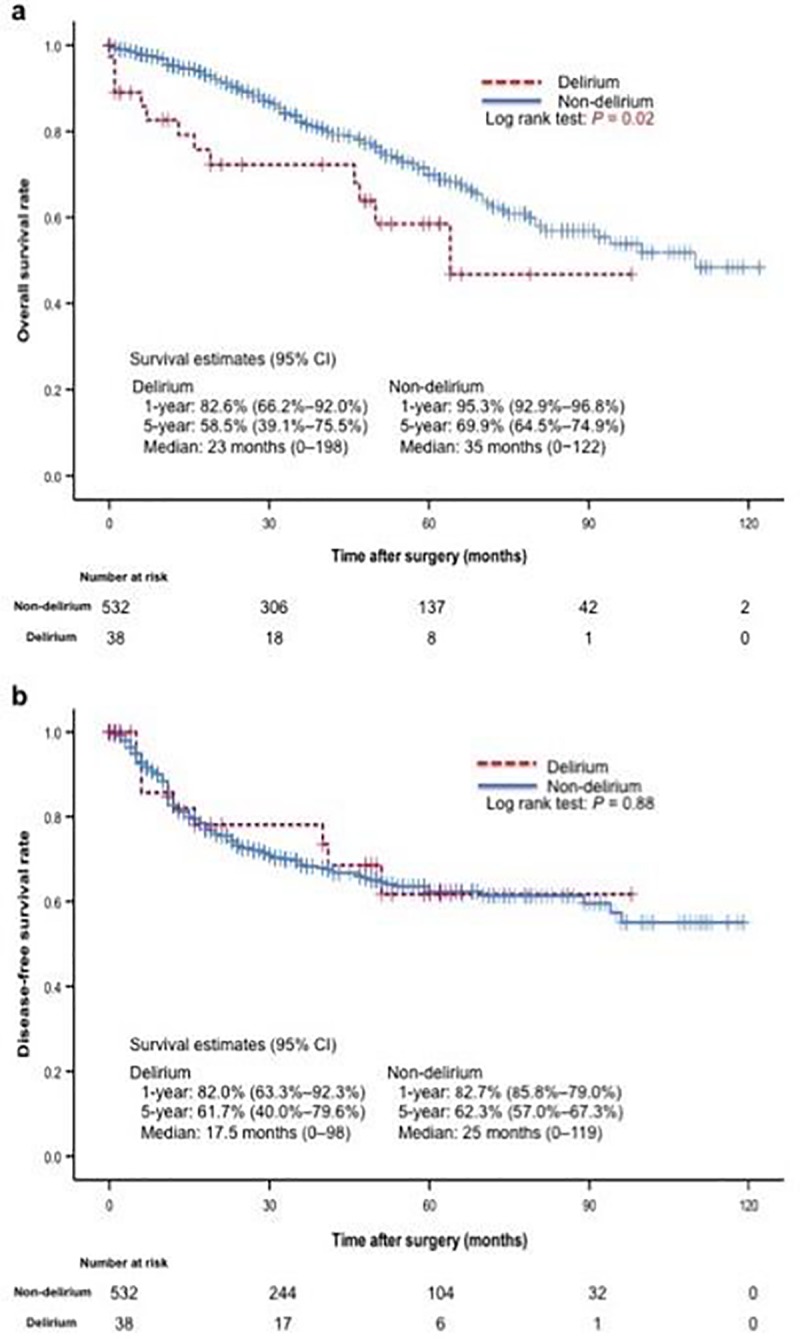
**Kaplan–Meier Curves for Overall Survival (a) and Disease-Free Survival (b) for the Delirium Group and the Non-delirium Group.** Although the overall survival rate was significantly lower in the delirium group, there was no significant difference between the two groups in the disease-free survival period. CI, confidence interval.

The median follow-up period was 35 months (range, 0–122 months). OS was significantly shorter in the delirium group (Fig 3A). There was no significant difference in DFS between the groups (Fig 3B). Among the 135 patients who died during the observation period, 13 had postoperative delirium. No significant changes were observed in the frequency of cancer-related deaths as compared to non-cancer related deaths between the delirium and non-delirium groups (Table 7).

**Table 7:** Causes of death.

Variable	Overall (n = 135)	Delirium (n = 13)	Non-delirium (n = 122)	*P* value
Lung cancer-specific death, n (%)	84 (62.2)	8 (61.5)	76 (62.3)	0.29^†^
Death from other cause, n (%)	51 (37.8)	5 (38.5)	46 (37.7)	0.66^†^

†,Log rank test

The purpose of this study was to clarify the risk factors of delirium occurring after primary lung cancer surgery, to construct a useful scoring system that can predict delirium, and to clarify the relationship between delirium and prognosis. There have been few reports discussing the risk of delirium occurring after primary lung cancer surgery. In our study, the risk factors that affected the onset of delirium after primary lung cancer surgery were "cerebrovascular disease history," "squamous cell carcinoma," and "age older than 75 years." Among them, the effect of "cerebrovascular disease history" was relatively larger. It has been reported that cerebrovascular disease history is a risk factor of delirium developing after hepatectomy [7]. Although not a postoperative factor, it was reported that history of cerebral infarction is a risk factor of delirium occurring in patients with heart failure [23]. It has been reported that abnormal oxygen metabolism in the brain due to hypoxia is a risk factor of delirium [24, 25]. From the past reports and our analysis, it is clear that history of cerebral ischemia could be a risk factor of developing delirium. It was reported that the presence of cerebral white matter lesions is a risk factor of delirium sharing the same mechanism with that of cerebral ischemia [17]. However, the mechanism by which past history of cerebral ischemia influences current delirium is unclear and we expect future neurophysiological elucidation.

Squamous-cell carcinoma was a second independent risk factor. Although several studies have examined delirium after pulmonary malignant-tumor surgery, detailed reports on the histologic structure have not been conducted [14,16]. In esophageal cancer, difference in histology was not a significant factor of postoperative delirium [26]. One potential reason for this is that in esophageal cancer, the number of patients with squamous-cell carcinoma is overwhelmingly larger than that of patients with adenocarcinoma. Since the occurrence of squamous-cell carcinoma involves a high degree of smoking, the effect of smoking may potentially contribute to the onset of postoperative delirium. In this study, based on univariate analysis, the patient group who developed delirium postoperatively had significantly higher Brinkman index, which is defined as the number of cigarettes smoked per day times the smoking years. Compared to other patients, patients with squamous-cell carcinoma may be more affected by oxygen metabolism and have a higher propensity to develop delirium after surgery, similar to the trend seen in cerebrovascular disorder. Focusing on the results of the respiratory function tests, there was a significant difference in %vital capacity alone, but the median of all items fluctuated within the normal range. This is considered to be due to the selection bias of treating only operable patients.

Older age was the third risk factor, which has also been reported by previous studies. In past reports, older age was a risk factor of postoperative delirium even in analysis using patient groups excluding patients with dementia [27]. This result suggests that delirium may not be caused simply by a clinically-significant decline in cognitive function. It has been proposed that the influence of respiratory and metabolic changes accompanying age-related changes contributes to the onset of postoperative delirium [24].

When comparing the postoperative course of the groups with and without delirium, no difference in mean length of ICU stay was observed. This was likely because patients in a stable condition were routinely discharged from the ICU on postoperative day 1. In this study, the time of onset of postoperative delirium was earlier compared to that in past reports [5,14]. One potential reason for this is that most patients entered the ICU after surgery and received intensive management. As such, diagnosis of delirium based on the CAM-ICU was often performed on the day of surgery. Although there was no significant difference in length of postoperative hospital days, the stay tended to be longer in the delirium group than in the non-delirium group (mean, 14.3 days versus 11.7 days, *P* = 0.96). One potential reason that a significant difference was not observed is that in Japan, there is a tendency for patients to be hospitalized for as long as they wish [28]. Therefore, it is conceivable that there were a number of cases in which the hospitalization period was prolonged without postoperative complications.

The frequency of postoperative complications and 30-day postoperative mortality were higher in the delirium group. Univariate analysis revealed that the patients in the delirium group were older and more likely to be heavy smokers, as well as to have preoperative hypoalbuminemia, high preoperative C-reactive protein, and high preoperative mean corpuscular volume than were the patients in the non-delirium group. These findings suggest that patients with delirium may have been in poor general condition compared with patients without delirium. Although no analysis was performed for each individual postoperative complication, the increased number of postoperative complications and 30-day postoperative mortality among patients who developed delirium may reflect the vulnerability of this group after surgery.

In the present study, OS was found to be shorter among patients who developed delirium after surgery. However, there was no significant difference in DFS, lung-cancer stage, or surgical procedure between the two groups. Among the 135 patients who died during the observation period, no differences in cause of death were observed between the groups with respect to death from cancer or other diseases. Therefore, patients who developed delirium after surgery were not more likely to die from cancer; indeed, other diseases were observed to be prevalent causes of death in both groups.

A previous study showed that OS was shorter in patients with squamous-cell carcinoma compared with patients with adenocarcinoma, although there was no difference in DFS between the groups [29]. This finding is similar to the results of the present study regarding patients who developed postoperative delirium. Patients with squamous-cell carcinoma were also older than those with adenocarcinoma, were more likely to smoke, and had more comorbidities. Kawase et al. concluded that death among patients with squamous-cell carcinoma was frequently attributable to these factors and not to the actual presence of lung cancer [29]. The same principle may apply to patients who develop delirium after surgery. In other words, shorter OS in patients who developed delirium after surgery for primary lung cancer may not be the result of delirium.

To date, this is the largest longitudinal study on delirium after lung resection for primary lung cancer. It is also the first study to examine survival and recurrence of lung cancer among patients who developed delirium after surgery for primary lung cancer.

This study has some limitations. Because in several cases oral medication was inadequately recorded, the effects of drugs, which have been previously reported as risk factors for delirium after surgery, were not evaluated [26,30]. As this was a single-center study of a unique population, the results may not be representative of the general population. In addition, the disproportionate number of delirium and non-delirium cases may have affected the robustness of the regression model. It should also be noted that, unfortunately, none of the risk factors identified in this study could be preoperatively improved. Therefore, future prospective studies are needed on effective interventions for the prevention of delirium after primary lung cancer surgery.

## Conclusions

In conclusion, we have clarified the risk factors of delirium after primary lung cancer surgery and succeeded in proposing a useful scoring system predicting the onset of delirium. Moreover, it was also found that the prognosis of patients with lung cancer who developed delirium after surgery was poor. Future studies are required to verify the results of this study. Our findings also indicate that the development of delirium after surgery for primary lung cancer may reflect patient vulnerability, and a prognostic factor among this patient population may not be lung cancer per se. Therefore, in order to improve the survival rate of these patient groups, follow up by a thoracic surgeon alone is insufficient; close observation and cooperation among various medical departments is crucial.

## Supporting information

S1 Dataset This is the raw patient data in this paper. (XLSX)
