# Supplementary material for: Postoperative delirium after lung resection for primary lung cancer: Risk factors, risk scoring system, and prognosis
Source: PLoS One. 2019 Nov 18;14(11):e0223917. doi: 10.1371/journal.pone.0223917 (PMC6860435; doi:10.1371/journal.pone.0223917)
Supplement: Attachment — Submitted filename: Response_to_Reviewers.docx [file pone.0223917.s002.docx]

September 24, 2019

Dr. Hyun-Sung Lee
Academic Editor
*PLOS ONE*

PONE-D-19-19575

Dr. Hyun-Sung Lee:

Please find attached a revised version of our manuscript “Postoperative delirium after lung resection for primary lung cancer: risk factors, risk scoring system, and prognosis,” which we would like to resubmit for publication in *PLOS ONE*.

Your comments and those of the reviewers were highly insightful and enabled us to greatly improve the quality of our manuscript. In the following pages are our point-by-point responses to each of the comments of the reviewers as well as to your own comments.

Revisions in the text are shown using yellow highlight for additions, and strikethrough *[~~example~~]* for deletions. In accordance with the reviewers’ suggestions, we have added details regarding the conditions at the time of stent placement and the progress after stent placement. We have also added and replaced some images. We hope that the revisions in the manuscript and our accompanying responses will be sufficient to make our manuscript suitable for publication in *PLOS ONE*.

We shall look forward to hearing from you at your earliest convenience.

Yours sincerely,

Kazuki Hayashi

Division of General Thoracic Surgery, Department of Surgery, Shiga University of Medical Science, Otsu, Japan

TEL: +81 77 548 2244

FAX: +81 77 544 2901

E-mail: hayashik@belle.shiga-med.ac.jp

**Responses to the comments of the Reviewers**

Reviewer #1: The authors conducted the paper entitled “Postoperative delirium after lung resection for primary lung cancer: risk factors, risk scoring system, and prognosis”.

This retrospective study describes the risk factors of postoperative delirium after pulmonary resection. Postoperative delirium is a serious issue owing to a growing number of pulmonary resection for geriatric and attracts the reader’s interests. However, what the authors mean in this paper lacks persuasion owing to some flaw. I have following comments to make after going through it.

Too many factors (pre-, intra-, postoperative information) were analyzed in this paper. I believe confounding bias existed (ex. smoking, COPD, and Squamous cell carcinoma). I believe factors should be confined depending on the number of cases (only 38 events out of 570 cases). Actually, histologic type should be excluded in this analysis.

***Response****: Certainly, this study analyzes many factors, most of which involve univariate analysis of patient background parameters. Histologic type is an important factor in discussing prognosis and is considered difficult to exclude. We have removed the passage concerning lung emphysematous changes from the Data Collection section (lines 82–84 to avoid confounding bias.*

Reviewer #2: This paper studied the risk factors of postoperative delirium. With increasing age postoperative complications including delirium are becoming a major concern. Delirium is problematic with regards to medical safety, longer hospital stays, and the need for additional medical attention.
This study established a scoring system for delirium, with the scores indicating the risk of postoperative delirium. The most important point raised in the paper is preventing postoperative delirium. If a patient requiring surgery has a high risk of delirium, what should be carried out prior to surgery?

***Response****: Unfortunately, none of the risk factors identified in this study could be improved by preoperative intervention. Therefore, we believe that what should be done for high-risk patients should be clarified in future prospective studies. This reasoning has been added to the text.*

Although overall survival of the patients who developed delirium was shorter than those who did not, delirium was not related with non-cancer specific death. This finding suggests that delirium is not related to the vulnerability of patients. The conclusion of the paper is therefore incorrect. In addition, DFS and OS was calculated by the Kaplan–Meier method. Cancer-specific survival or non-cancer-specific survival should therefore be calculated using the same method.

***Response****: When comparing patients with and without delirium, the results showed that the OS was shorter in patients with delirium, even though there was no significant difference in DFS. In addition, there was no difference in the cause of death between the two groups. This suggests that the presence or absence of delirium and the malignancy of the cancer may be unrelated. Therefore, when considering the reason why the OS of delirium patients was significantly shorter, we have referred to the findings of a previous paper that compared squamous cell carcinoma with adenocarcinoma.. However, as you indicated, some of the descriptions regarding the vulnerabilities in survival have been deleted because of lack of logical support. However, it is clear from the patient background comparison that the patients who developed delirium were more physically vulnerable, as stated in the Discussion. The table content has been modified to cancer-specific survival and non-cancer-specific survival calculated using the Kaplan–Meier method (Table 6).*

The 30-day mortality rate appears to be higher (14 of 570; 2.5%). The annual report of the Japanese Association for Thoracic Surgery (Gen Thorac Cardiovasc Surg 2019; 67:377–411) reported that 30-day mortality rate was 0.4%. This high mortality rate after lung cancer surgery may have affected the results of this study. Please describe the causes of mortality in detail.

***Response****: The 30-day mortality rate was 1.2% (7 cases), not 2.5% (14 cases). The causes of death of these 7 cases has been tabulated (Table 3b).*

Postoperative complications were related with delirium (Table 3). Accordingly, postoperative recovery would affect the development of delirium. However, analyses of the postoperative factors were not sufficient. The enhanced recovery after surgery (ERAS) program is considered to be effective for avoiding postoperative complications. There is no information in the paper about early ambulation and perioperative management.

***Response****: I understand, but there was no active ERAS program at our hospital during the observation period of this study. In addition, postoperative rehabilitation varies depending on the physician in charge. Therefore, we could not uniformly discuss perioperative management in this study.*

In the patient demographics, “pulmonary emphysematous change” is obscure and should be changed to pulmonary emphysema or COPD.

***Response****: I have removed the “emphysematous change” item.*

In line 229, the authors discussed the relationship between ICU stay and delirium. Did all the patients stay in the ICU? The rate of ICU admissions would be different between institutions. It is not a problem where the patients stay after surgery?

***Response****: With the exception of wedge resection, most patients enter the ICU. As you mentioned, ICU admission rates are considered to be large among facilities. However, as listed in the references, some previous studies have discussed postoperative delirium and ICU admission, and we included information of ICU admission for comparative purposes*

Reviewer #3: This is a single institution, retrospective cohort study that examines 570 patients with a history of primary lung cancer surgery in 2006 and 2017 designed to determine the risk factors related to the onset of postoperative delirium, to construct a clinically useful scoring system, and to investigate the relationship between the onset of delirium and prognosis. You have developed a new scoring system with three variables including old age, cerebrovascular disease history, and squamous cell carcinoma to predict postoperative delirium. And, you have demonstrated that the prognosis of patients with lung cancer who developed delirium after surgery was unfavorable. You have suggested that multidisciplinary approaches to take care of patients who undergo lung cancer surgery are required to improve the survival rate.

1. You should clarify how to define postoperative delirium since your study period looks so long compared with years in cited references.

***Response****: As you indicated, the DSM-5 was published after the first half of the observation period; thus, we diagnosed postoperative delirium using the DSM-IV-TR or DSM-5 as available at the time of enrolment.*

2. At a glance, old, male, smokers with squamous cell carcinoma seem to have high risk for the development of postoperative dementia. All three variables in your scoring system are unmodifiable factors. I am not sure how much the prediction of postoperative dementia is helpful to improve patient outcomes through multidisciplinary approaches. Please provide your detailed suggestion on its clinical application. Modifiable factors to have potential to be associated with the development of delirium would be more interesting.

***Response****: As you said, unfortunately, none of the risk score items can be improved. I am somewhat disappointed by this result because improving modifiable factors could help prevent postoperative delirium. We plan to investigate delirium prevention in future studies, such as in clinical trials of preventive drug administration. I have added this information to the revised manuscript.*
3. Since the number of events is only 38, you would be better to use the penalized maximum estimation method, so called Firth regression method, to generate regression coefficients from multivariable analysis. Please describe how to select variables for multivariable analysis among many factors that are significant in univariable analysis. And, perform multicollinearity test to reduce bias.

***Response****: Unfortunately, using the Firth regression method you indicated was not possible with my statistical software. The stepwise method is not generally recommended for variable selection and was not used. The number of events was 38, and three items that were considered important in past reports and of interest for this patient group were selected. In the multicollinearity test, the correlation coefficient between age and tissue type was 0.084, the correlation coefficient between age and cerebrovascular disorder was 0.072, and the correlation coefficient between tissue type and cerebrovascular disorder was 0.028.*

4. You would be better to provide the quality of the prediction model using several model performance measures, including calibration and decision curve analysis.

***Response****: We would appreciate the Reviewer’s thoughts on whether and/or why the described Hosmer–Lemeshow test results would be considered insufficient.*
