# Supplementary material for: Postoperative delirium after lung resection for primary lung cancer: Risk factors, risk scoring system, and prognosis
Source: PLoS One. 2019 Nov 18;14(11):e0223917. doi: 10.1371/journal.pone.0223917 (PMC6860435; doi:10.1371/journal.pone.0223917)
Supplement: Peer review history

# Peer review: Decision Letter 0

**DOI:** 10.1371/journal.pone.0223917.r001

**Authors:** Hyun-Sung Lee

13 Aug 2019

PONE-D-19-19575

Postoperative delirium after lung resection for primary lung cancer: risk factors, risk scoring system, and prognosis

PLOS ONE

Dear Dr. Hayashi,

Thank you for submitting your manuscript to PLOS ONE. After careful consideration, we feel that it has merit but does not fully meet PLOS ONE’s publication criteria as it currently stands. Therefore, we invite you to submit a revised version of the manuscript that addresses the points raised during the review process.

Reviewers raised constructive comments. They pointed out the small number of events (postopertaive delirium). You would be better to provide more supportive evidence of you prediction model using advanced statistical methods. 

We would appreciate receiving your revised manuscript by Sep 27 2019 11:59PM. When you are ready to submit your revision, log on to https://www.editorialmanager.com/pone/ and select the 'Submissions Needing Revision' folder to locate your manuscript file.

If you would like to make changes to your financial disclosure, please include your updated statement in your cover letter.

To enhance the reproducibility of your results, we recommend that if applicable you deposit your laboratory protocols in protocols.io, where a protocol can be assigned its own identifier (DOI) such that it can be cited independently in the future. For instructions see: http://journals.plos.org/plosone/s/submission-guidelines#loc-laboratory-protocols

Please include the following items when submitting your revised manuscript:

- A rebuttal letter that responds to each point raised by the academic editor and reviewer(s). This letter should be uploaded as separate file and labeled 'Response to Reviewers'.
- A marked-up copy of your manuscript that highlights changes made to the original version. This file should be uploaded as separate file and labeled 'Revised Manuscript with Track Changes'.
- An unmarked version of your revised paper without tracked changes. This file should be uploaded as separate file and labeled 'Manuscript'.

Please note while forming your response, if your article is accepted, you may have the opportunity to make the peer review history publicly available. The record will include editor decision letters (with reviews) and your responses to reviewer comments. If eligible, we will contact you to opt in or out.

We look forward to receiving your revised manuscript.

Kind regards,

Hyun-Sung Lee, M.D., Ph.D.

Academic Editor

PLOS ONE

Journal Requirements:

1. When submitting your revision, we need you to address these additional requirements.

Please ensure that your manuscript meets PLOS ONE's style requirements, including those for file naming. The PLOS ONE style templates can be found at

http://www.journals.plos.org/plosone/s/file?id=wjVg/PLOSOne_formatting_sample_main_body.pdf and http://www.journals.plos.org/plosone/s/file?id=ba62/PLOSOne_formatting_sample_title_authors_affiliations.pdf

2. Please amend your current ethics statement to address the following concerns:  

a) Did participants provide their written or verbal informed consent to participate in this study?

b) If consent was verbal, please explain i) why written consent was not obtained, ii) how you documented participant consent, and iii) whether the ethics committees/IRB approved this consent procedure.

[Note: HTML markup is below. Please do not edit.]

Reviewers' comments:

Reviewer's Responses to Questions

**Comments to the Author**

1. Is the manuscript technically sound, and do the data support the conclusions?

The manuscript must describe a technically sound piece of scientific research with data that supports the conclusions. Experiments must have been conducted rigorously, with appropriate controls, replication, and sample sizes. The conclusions must be drawn appropriately based on the data presented. 

Reviewer #1: Partly

Reviewer #2: Yes

Reviewer #3: Partly

**********

2. Has the statistical analysis been performed appropriately and rigorously? 

Reviewer #1: N/A

Reviewer #2: Yes

Reviewer #3: No

**********

3. Have the authors made all data underlying the findings in their manuscript fully available?

The PLOS Data policy requires authors to make all data underlying the findings described in their manuscript fully available without restriction, with rare exception (please refer to the Data Availability Statement in the manuscript PDF file). The data should be provided as part of the manuscript or its supporting information, or deposited to a public repository. For example, in addition to summary statistics, the data points behind means, medians and variance measures should be available. If there are restrictions on publicly sharing data—e.g. participant privacy or use of data from a third party—those must be specified.

Reviewer #1: Yes

Reviewer #2: Yes

Reviewer #3: Yes

**********

4. Is the manuscript presented in an intelligible fashion and written in standard English?

PLOS ONE does not copyedit accepted manuscripts, so the language in submitted articles must be clear, correct, and unambiguous. Any typographical or grammatical errors should be corrected at revision, so please note any specific errors here.

Reviewer #1: Yes

Reviewer #2: Yes

Reviewer #3: Yes

**********

5. Review Comments to the Author

Please use the space provided to explain your answers to the questions above. You may also include additional comments for the author, including concerns about dual publication, research ethics, or publication ethics. (Please upload your review as an attachment if it exceeds 20,000 characters)

Reviewer #1: The authors conducted the paper entitled “Postoperative delirium after lung resection for primary lung cancer: risk factors, risk scoring system, and prognosis”.

This retrospective study describes the risk factors of postoperative delirium after pulmonary resection. Postoperative delirium is a serious issue owing to a growing number of pulmonary resection for geriatric and attracts the reader’s interests. However, what the authors mean in this paper lacks persuasion owing to some flaw. I have following comments to make after going through it.

Too many factors (pre-, intra-, postoperative information) were analyzed in this paper. I believe confounding bias existed (ex. smoking, COPD, and Squamous cell carcinoma). I believe factors should be confined depending on the number of cases (only 38 events out of 570 cases). Actually, histologic type should be excluded in this analysis.

Reviewer #2: This paper studied the risk factors of postoperative delirium. With increasing age postoperative complications including delirium are becoming a major concern. Delirium is problematic with regards to medical safety, longer hospital stays, and the need for additional medical attention.

This study established a scoring system for delirium, with the scores indicating the risk of postoperative delirium. The most important point raised in the paper is preventing postoperative delirium. If a patient requiring surgery has a high risk of delirium, what should be carried out prior to surgery?

Although overall survival of the patients who developed delirium was shorter than those who did not, delirium was not related with non-cancer specific death. This finding suggests that delirium is not related to the vulnerability of patients. The conclusion of the paper is therefore incorrect. In addition, DFS and OS was calculated by the Kaplan–Meier method. Cancer-specific survival or non-cancer-specific survival should therefore be calculated using the same method.

The 30-day mortality rate appears to be higher (14 of 570; 2.5%). The annual report of the Japanese Association for Thoracic Surgery (Gen Thorac Cardiovasc Surg 2019; 67:377–411) reported that 30-day mortality rate was 0.4%. This high mortality rate after lung cancer surgery may have affected the results of this study. Please describe the causes of mortality in detail.

Postoperative complications were related with delirium (Table 3). Accordingly, postoperative recovery would affect the development of delirium. However, analyses of the postoperative factors were not sufficient. The enhanced recovery after surgery (ERAS) program is considered to be effective for avoiding postoperative complications. There is no information in the paper about early ambulation and perioperative management.

In the patient demographics, “pulmonary emphysematous change” is obscure and should be changed to pulmonary emphysema or COPD.

In line 229, the authors discussed the relationship between ICU stay and delirium. Did all the patients stay in the ICU? The rate of ICU admissions would be different between institutions. It is not a problem where the patients stay after surgery?

Reviewer #3: This is a single institution, retrospective cohort study that examines 570 patients with a history of primary lung cancer surgery in 2006 and 2017 designed to determine the risk factors related to the onset of postoperative delirium, to construct a clinically useful scoring system, and to investigate the relationship between the onset of delirium and prognosis. You have developed a new scoring system with three variables including old age, cerebrovascular disease history, and squamous cell carcinoma to predict postoperative delirium. And, you have demonstrated that the prognosis of patients with lung cancer who developed delirium after surgery was unfavorable. You have suggested that multidisciplinary approaches to take care of patients who undergo lung cancer surgery are required to improve the survival rate.

1. You should clarify how to define postoperative delirium since your study period looks so long compared with years in cited references.

2. At a glance, old, male, smokers with squamous cell carcinoma seem to have high risk for the development of postoperative dementia. All three variables in your scoring system are unmodifiable factors. I am not sure how much the prediction of postoperative dementia is helpful to improve patient outcomes through multidisciplinary approaches. Please provide your detailed suggestion on its clinical application. Modifiable factors to have potential to be associated with the development of delirium would be more interesting.

3. Since the number of events is only 38, you would be better to use the penalized maximum estimation method, so called Firth regression method, to generate regression coefficients from multivariable analysis. Please describe how to select variables for multivariable analysis among many factors that are significant in univariable analysis. And, perform multicollinearity test to reduce bias.

4. You would be better to provide the quality of the prediction model using several model performance measures, including calibration and decision curve analysis.

**********

6. PLOS authors have the option to publish the peer review history of their article (what does this mean?). If published, this will include your full peer review and any attached files.

If you choose “no”, your identity will remain anonymous but your review may still be made public.

**Do you want your identity to be public for this peer review?** For information about this choice, including consent withdrawal, please see our Privacy Policy.

Reviewer #1: No

Reviewer #2: Yes: Satoshi Shiono

Reviewer #3: No

[NOTE: If reviewer comments were submitted as an attachment file, they will be attached to this email and accessible via the submission site. Please log into your account, locate the manuscript record, and check for the action link "View Attachments". If this link does not appear, there are no attachment files to be viewed.]

While revising your submission, please upload your figure files to the Preflight Analysis and Conversion Engine (PACE) digital diagnostic tool, https://pacev2.apexcovantage.com/. PACE helps ensure that figures meet PLOS requirements. To use PACE, you must first register as a user. Registration is free. Then, login and navigate to the UPLOAD tab, where you will find detailed instructions on how to use the tool. If you encounter any issues or have any questions when using PACE, please email us at figures@plos.org. Please note that Supporting Information files do not need this step.

# Author comment: Author response to Decision Letter 0

**DOI:** 10.1371/journal.pone.0223917.r002

26 Sep 2019

Your comments and those of the reviewers were highly insightful and enabled us to greatly improve the quality of our manuscript.

Attachment Submitted filename: Response_to_Reviewers.docx Click here for additional data file.

# Editor report: Decision Letter 1

**DOI:** 10.1371/journal.pone.0223917.r003

**Authors:** Hyun-Sung Lee

2 Oct 2019

Postoperative delirium after lung resection for primary lung cancer: risk factors, risk scoring system, and prognosis

PONE-D-19-19575R1

Dear Dr. Hayashi,

We are pleased to inform you that your manuscript has been judged scientifically suitable for publication and will be formally accepted for publication once it complies with all outstanding technical requirements.

Within one week, you will receive an e-mail containing information on the amendments required prior to publication. When all required modifications have been addressed, you will receive a formal acceptance letter and your manuscript will proceed to our production department and be scheduled for publication.

Shortly after the formal acceptance letter is sent, an invoice for payment will follow. To ensure an efficient production and billing process, please log into Editorial Manager at https://www.editorialmanager.com/pone/, click the "Update My Information" link at the top of the page, and update your user information. If you have any billing related questions, please contact our Author Billing department directly at authorbilling@plos.org.

If your institution or institutions have a press office, please notify them about your upcoming paper to enable them to help maximize its impact. If they will be preparing press materials for this manuscript, you must inform our press team as soon as possible and no later than 48 hours after receiving the formal acceptance. Your manuscript will remain under strict press embargo until 2 pm Eastern Time on the date of publication. For more information, please contact onepress@plos.org.

With kind regards,

Hyun-Sung Lee, M.D., Ph.D.

Academic Editor

PLOS ONE

Additional Editor Comments (optional):

Reviewers' comments:

# Editor report: Acceptance letter

**DOI:** 10.1371/journal.pone.0223917.r004

**Authors:** Hyun-Sung Lee

8 Nov 2019

PONE-D-19-19575R1 

Postoperative delirium after lung resection for primary lung cancer: risk factors, risk scoring system, and prognosis 

Dear Dr. Hayashi:

I am pleased to inform you that your manuscript has been deemed suitable for publication in PLOS ONE. Congratulations! Your manuscript is now with our production department. 

If your institution or institutions have a press office, please notify them about your upcoming paper at this point, to enable them to help maximize its impact. If they will be preparing press materials for this manuscript, please inform our press team within the next 48 hours. Your manuscript will remain under strict press embargo until 2 pm Eastern Time on the date of publication. For more information please contact onepress@plos.org.

For any other questions or concerns, please email plosone@plos.org. 

Thank you for submitting your work to PLOS ONE.

With kind regards,

PLOS ONE Editorial Office Staff

on behalf of

Dr. Hyun-Sung Lee 

Academic Editor

PLOS ONE
